# Activated Carbons Obtained from Orange Peels, Coffee Grounds, and Sunflower Husks—Comparison of Physicochemical Properties and Activity in the Alpha-Pinene Isomerization Process

**DOI:** 10.3390/ma14237448

**Published:** 2021-12-04

**Authors:** Adrianna Kamińska, Piotr Miądlicki, Karolina Kiełbasa, Marcin Kujbida, Joanna Sreńscek-Nazzal, Rafał Jan Wróbel, Agnieszka Wróblewska

**Affiliations:** Department of Catalytic and Sorbent Materials Engineering, Faculty of Chemical Technology and Engineering, West Pomeranian University of Technology in Szczecin, Piastów Ave. 42, 71-065 Szczecin, Poland; kaminska.adrianna@zut.edu.pl (A.K.); piotr.miadlicki@zut.edu.pl (P.M.); Karolina.Kielbasa@zut.edu.pl (K.K.); marcin.kujbida@zut.edu.pl (M.K.); rafal.wrobel@zut.edu.pl (R.J.W.)

**Keywords:** isomerization, alpha-pinene, biomass, activated carbon, camphene, limonene

## Abstract

This work presents studies on the preparation of porous carbon materials from waste biomass in the form of orange peels, coffee grounds, and sunflower seed husks. The preparation of activated carbons from these three waste materials involved activation with KOH followed by carbonization at 800 °C in an N_2_ atmosphere. This way of obtaining the activated carbons is very simple and requires the application of only two reactants. Thus, this method is cheap, and it does not generate much chemical waste. The obtained activated carbons were characterized by XRD, SEM, XPS, and XRF methods. Moreover, the textural properties, acidity, and catalytic activity of these materials were descried. During catalytic tests carried out in the alpha-pinene isomerization process (the use of the activated carbons thus obtained in the process of alpha-pinene isomerization has not been described so far), the most active were activated carbons obtained from coffee grounds and orange peels. Generally, the catalytic activity of the obtained materials depended on the pore size, and the most active activated carbons had more pores with sizes of 0.7–1.0 and 1.1–1.4 nm. Moreover, the presence of potassium and chlorine ions in the pores may also be of key importance for the alpha-pinene isomerization process. On the other hand, the acidity of the surface of the tested active carbons did not affect their catalytic activity. The most favorable conditions for carrying out the alpha-pinene isomerization process were the same for the three tested activated carbons: temperature 160 °C, amount of the catalyst 5 wt.%, and reaction time 3 h. Kinetic studies were also carried out for the three tested catalysts. These studies showed that the isomerization over activated carbons from orange peels, coffee grounds, and sunflower seed husks is a first-order reaction.

## 1. Introduction

The synthesis of activated carbons from biomass has become an interesting way to obtain useful carbonaceous materials from raw materials that are a waste byproduct of the food industry. In recent years, carbon materials have become functional materials used on a large scale in many processes [1]. The problem with biowaste, which is constantly increasing, is the cost of the utilization of these materials [2]. The use of waste biomass, whose disposal is difficult and costly, can have a positive impact on the environment. The advantages of activated carbons obtained from biomass include efficiency and low production costs compared to commercial activated carbons [3]. Currently, materials of natural origin are often used as precursors in the synthesis of carbonaceous materials [4]. For this purpose are used, e.g., corn cobs [5], nutshells [6], pomegranate peels [7], coconut shells [8], coir pith [9], brazilian nutshell [10], palm fruits shells [11], oil palm fruits shells [12], olive stones [13], jackfruit shell waste and jackfruit peels [14], rice husk [15], banana peels [16], apple pulp [17], cotton stalk [18], and egg white biomass [19]. There are also reports describing biological precursors used in the production of carbonaceous materials [20].

One of the waste products used as precursors for the synthesis of activated carbons are orange peels. The citrus processing industry generates huge amounts of orange peels annually, which are the byproduct of the industrial extraction of citrus juices and waste from the production of jams and marmalades [21]. Orange juice is currently one of the most frequently chosen drinks by consumers around the world [22]. When oranges are processed into other food products, solid residues (peel, flesh, and seeds) and liquid waste, so-called “yellow water”, are formed [23]. All of these wastes are composed of cellulose, hemicellulose, proteins, essential oils, pectins, sugars, organic acids, and salt [24]. During the treatment of this biowaste, it is necessary to neutralize the pH of the biomass, which is a serious problem for industries dealing with disposal [25]. This biomass contains large amounts of organic matter; therefore, it can pollute the environment if it is improperly stored and disposed of in an improper way [26].

Activated carbons produced from waste orange peels are used as adsorbents to remove organic compounds from the natural environment, e.g., naphthalene and 1-naphthol [27], dyes and chlorophenoxyacetic acid herbicide [28], for obtaining cathodes for the production of capacitors [29,30], and as catalysts (doped with TiO_2_) for NO_X_ decomposition [31]. Moreover, carbonaceous materials obtained from orange peels are used as catalysts in the esterification of oleic acid and citric acid [32] and, after modification with iron ions, as SO_2_ sorbents [33]. A method for the preparation of selective and sensitive electrochemical sensors, which are used for the detection of toxic metal ions, was also described. In this method, palladium nanoparticles were deposited on the porous activated carbon obtained from orange peels [34].

Coffee is the second most consumed drink after water. Worldwide, about 500 billion cups are consumed annually, which equates to about nine million tons of waste from coffee grounds [35]. Coffee ground waste consists of caffeine, cellulose, hemicellulose, polysaccharides, and lignin, which, if not properly stored and disposed, can cause environmental pollution [36]. One of the ways to use coffee ground waste can be its application as a material for the production of activated carbons.

Activated carbons from coffee grounds are used as organic dye adsorbents [37], organic compounds adsorbents [38], and CO_2_ adsorbents [39]. Carbonaceous materials from coffee grounds are also used as the catalysts in many organic reactions, e.g., glycerol etherification [40], sulfamethoxazole degradation [41], and oleic acid and methanol esterification [42]. These materials also found applications in electrochemistry as anodes for lithium-ion batteries [43], as electrocatalysts [44], and as materials for energy storage [45].

In recent years, the importance of sunflower as an oil plant has increased. For years, both the production and the consumption of oils have steadily increased. The total annual world production of sunflower is about 20.9 million tons of seeds from about 18 million hectares of arable land [3]. Taking into account that sunflower husks constitute 21–30% of the total weight of seeds and are the waste byproduct, appropriate methods should be developed that allow for environmentally friendly applications of this waste material [46]. The sunflower husk waste contains a significant amount of organic matter (proteins, cellulose, hemicelluloses, and lignin) that requires proper disposal and storage [47]. There are some reports in the scientific literature of sunflower husks used in the production of carbonaceous materials.

Carbonaceous materials obtained from waste sunflower found applications in the adsorption of Acid Violet 17 (AV17) [48], dyes (methylene blue and acid blue) [49], copper ions from wastewater [50], and phenol from aqueous solutions [51].

According to the current trends in science, the aim is to obtain high-value-added products from relatively cheap substrates, including alpha-pinene. Alpha-pinene is a cyclic organic and monoterpene compound [52]. This compound is obtained by a distillation of turpentine obtained from pine trees and is also a cheap raw material for many organic reactions [53]. Alpha-pinene is a commercially significant organic compound as it is the raw material for the synthesis of high-value-added products that are used in many industrial sectors (perfumery, cosmetics, food, and pharmaceuticals) [54,55]. 

The alpha-pinene isomerization reaction allows obtaining very important organic compounds, such as camphene and limonene. Both of these compounds are mainly used in cosmetics [56] and perfumery [57]. In addition, camphene is used as the raw material in obtaining insecticides [58] and as a pyrogen to produce nano/macroporous polycaprolactone [59]. However, the most common application of this compound is the production of synthetic camphor [57].

Limonene is an organic compound present in orange oil, which is produced from orange peels, a waste biomass from the orange juice industry [60,61]. Limonene is used as the raw material for obtaining many intermediates in laboratory organic chemistry and in the chemical industry, as well as a solvent for reactions performed with organic compounds, because it is not only cheap, but also biodegradable and not very toxic [62]. Limonene is also used as an aroma in food and drinks, as well as a fragrance in air fresheners. In the cosmetics industry, limonene is used as an ingredient in many cosmetics, including creams [63].

Finding the appropriate catalyst with high catalytic activity and selectivity to the alpha-pinene isomerization process is still difficult. In large-scale production, alpha-pinene is produced using titanium dioxide as the catalyst. The main disadvantages of this method are long reaction time, high process temperature, and low selectivity to bicyclic compounds [64].

Currently, the isomerization of alpha-pinene in the presence of catalysts is the subject of many laboratory-scale studies. The catalysts which are mainly used in the isomerization of alpha-pinene are acid-modified materials (HCl-modified clinoptilolite [65], H_2_SO_4_-modified clinoptilolite [66], acid-modified illite [67]), materials modified with metal ions [68] (Fe-loaded clinoptilolite [69], Al-MCM-41 [70], Ti-MCM-41 [71], Ti-SBA-15 [72], Al-SBA-15 [73], and Ga-SBA-15 [74]), calcined natural zeolites [75,76] natural zeolite [77], W_2_O_3_–Al_2_O_3_ catalysts [78], phosphotungstic heteropoly acids [79], acidic functionalized ionic liquids [80], mesoporous molecular sieves [81] and sulfated zirconia catalysts [82]. Table 1 compares the activity of the selected catalysts used in the process of alpha-pinene isomerization.

Considering the amount of biowaste produced in the form of orange peels, sunflower husks, and coffee grounds, as well as the problem of their storage and environmentally safe disposal, we should strive to develop very effective and environmentally friendly biowaste management technologies. The method of synthesis of activated carbons, presented in this work by our team, allows application of the biomass in the form of orange peels, coffee grounds, or sunflower husks as materials (after carbonization) for conducting catalytic reactions (isomerization reaction). In this way, we can obtain valuable products from relatively inexpensive raw materials. According to current knowledge, catalysts based on biomass (orange peels, sunflower husks, and coffee grounds) have not been used so far in the alpha-pinene isomerization reaction.

The carbonization method presented by our team is a simple way to obtain activated carbons from waste biomass from the food industry. In comparison to the methods presented in the literature [28,30,34,39,45,51] the method of production of active carbons described in our work is very simple and it requires the application of only two chemical reactants, which makes it a very cheap method that generates little waste. The aim of this work was to obtain porous carbon materials from orange peels, coffee grounds, and sunflower seed husks and check their catalytic activity in the process of alpha-pinene isomerization. The production of activated carbons from waste materials involved chemical activation with KOH followed by carbonization at 800 °C in the atmosphere of N_2_. The aim of this stage was not only to obtain carbon materials, but also to characterize them using the following instrumental methods: XRD, SEM, XPS, and XRF. The textural properties of the tested materials were also described. Moreover, their acidity was determined. In the second stage, the catalytic activity of the obtained activated carbons was tested in the alpha-pinene isomerization process. The aim of this stage was to determine the best conditions for the isomerization process on the three tested catalysts. The aim of the last stage was to investigate the kinetics of the alpha-pinene isomerization process on catalyst samples tested in this work.

## 2. Materials and Methods

### 2.1. Preparation of Raw Materials of Natural Origin for the Process of Chemical Activation and Carbonization

In the studies, the following biomass materials were used to produce activated carbons: waste orange peels, coffee grounds, and sunflower husks.

The residual pulp was removed from the fresh orange peels. Coffee grounds and sunflower husks were washed several times with distilled water, and then each biomass was dried at ambient temperature for 24 h. After 24 h, the pre-dried biomass in the form of orange peels and sunflower husks was dried at 50 °C for another 24 h. On the other hand, the coffee grounds were dried at 70 °C, because drying at the lower temperature caused the rotting of this raw material and was ineffective. After the completing of drying, the raw materials were ground into a powder in a laboratory grinder.

### 2.2. Chemical Activation and Carbonization of Biomass

Chemical activation of biomass was carried out with the use of a saturated aqueous solution of potassium hydroxide. Biomass was weighed into a plastic cup, and then a KOH solution was added in such an amount that the weight ratio of biomass to activator was 1:1. The material was then vigorously mixed until the raw material was clearly saturated with KOH solution and left at ambient temperature for 3 h. After this time, the impregnated material was placed in a laboratory dryer (Alpina, Konin, Poland) (19 h, 200 °C). Next the carbonaceous substrate impregnated in this way was carbonized. The process of thermal decomposition of biomass was carried out in a tube furnace (Czylok, Jastrzębie-Zdrój, Poland) in an inert gas atmosphere (N_2_). The sample of biomass was heated to 800 °C for 1 h; then, the samples were kept at a given temperature for 1 h. After the carbonization process was completed, the sample was cooled to room temperature in a nitrogen atmosphere. The chemical activation and carbonization process were carried out in the same conditions and at the same stages for all waste materials.

The resulting carbon materials were named as follows:

O_AC—activated carbon from waste orange peels.

C_AC—activated carbon from coffee grounds.

S_AC—activated carbon from sunflower husks.

### 2.3. Characterization the Obtained Activated Carbons

The ASAP Sorption Surface Area and Pore Size Analyzer (ASAP 2460, Micrometrics, Norcross, GA, USA, 2018) was used for textural characterization of carbon samples. Nitrogen adsorption isotherms were measured at −196 °C. In order to measure the absorption, the samples were degassed at 250 °C for 19 h. The Brunauer–Emmett–Teller (BET) method was applied for calculation of the specific surface area. To estimate the value of the total pore volume (V_tot_), the adsorbed gas (nitrogen) volume was determined at a relative pressure of ~1. Micropore volumes were calculated by DFT (density functional theory) based on the N_2_ adsorption (slit N_2_-DFT Model adsorption). Through application of the DFT model in the ASAP 2460 version 3.01 software package based on nitrogen sorption isotherms, the pore size distribution in the tested materials was determined.

Scanning electron microscopy with cold field emission (SU8020 Ultrahigh-Resolution Field-Emission Scanning Electron Microscope; Hitachi Ltd., Hitachi City, Japan, 2012) was used to observe the morphology of the obtained carbon samples. The samples were prepared as follows: the material was ground and then placed on a double-sided conductive carbon tape, which was stuck to the SEM stub.

The X-ray diffraction (XRD) patterns of the catalysts were recorded by an X-ray diffractometer (X’Pert–PRO, Panalytical Almelo, The Netherlands, 2012) using Cu K_α_ (λ = 0.154 nm) as the radiation source in the 2θ range of 10–80° with a step size of 0.026.

The X-ray photoelectron spectroscopy measurements were performed in a commercial multipurpose (XPS, AES, LEED, UPS) ultrahigh vacuum (UHV) surface analysis system (PREVAC). The base pressure attainable in the system is low (10^−10^ mbar range). The UHV system consists of preparation and analysis chambers. The analysis chamber was equipped with a kinetic electron energy analyzer (SES-2002, Scienta Scientific AB, Uppsala, Sweden, 2002) and nonmonochromatic X-ray photoelectron spectroscope (XPS, PREVAC, Rogów, Poland, 2007). The calibration of the spectrometer was performed using Ag 3*d*_5/2_ transition. Samples in the form of powder were degassed under a vacuum for 1 day prior to measurements. The vacuum during XPS measurements was in the low range (10^−9^ mbar). The X-ray photoelectron spectroscopy was performed using Mg Ka radiation (hn = 1253.7 eV).

The titration method was used to determine the concentration of acid centers in the activated carbons obtained from biomass [66]. The method of the determination of acid centers consisted of the following steps: 20 mg of carbonaceous material was added to 10 cm^3^ of an aqueous solution of NaOH (0.01 M). Next, the mixture was placed in laboratory shaker at ambient temperature for 4 h. Then, the material was centrifuged. In the next stage, the supernatant solution of the activated carbon sediment was taken, and its pH was determined. The pH was established by titration with a 0.01 M aqueous solution of HCl. Phenolphthalein was used as an indicator. In order to calculate the concentrations of the acid centers (*Ns*) in the samples, the following formula was used:

$$Ns=\frac{\left({\left[OH^{-}\right]}_{0}^{}-{\left[OH^{-}\right]}_{4h}^{}\right)\times V}{m},$$

where [*OH*^−^] is the molar concentration of hydroxide groups determined by the titration method (mol/dm^3^), *V* is the volume of water solution of NaOH added to carbonaceous sample, and *m* is the mass of activated carbon sample.

In this work, in order to deepen the analysis of the results of catalytic tests, the XRF (X-ray fluorescence) method was also applied. The studies were carried out with the use of the Epsilon3 apparatus (Panalytical, Almelo, The Netherlands, 2011).

### 2.4. Alpha-Pinene Isomerization Method

The reaction of alpha-pinene isomerization was carried out in a glass reactor in which a reflux condenser was installed. The glass reactor was placed in an oil bath. During studies of the isomerization, 3 g of alpha-pinene (98%, Aldrich, St. Louis, MO, USA) and the appropriate amounts of activated carbons were used. The activity of carbonaceous materials was tested under the following conditions: reaction temperature 160 °C, catalyst amount 5 wt.%, reaction time 3 h, and speed of mixing 400 rpm. All activated carbons were next used to determine the most favorable reaction conditions. The influence of the following parameters was studied: temperature in the range of 140–180 °C, activated carbon content in the range of 1–5 wt.%, and reaction time from 10 min to 330 min.

The quantitative analyses of the post-reaction mixtures were performed by the gas chromatography method using a Thermo Electron FOCUS chromatograph with an FID detector and a ZB-1701 column (30 m × 0.53 mm × 1 μm, 14% cyanopropylphenyl, 86% dimethylpolysiloxane) (Anchem, Warszawa, Poland, 2009). The parameters of the chromatograph were as follows: helium flow 1.2 mL/min, injector temperature 220 °C, detector temperature 250 °C, furnace temperature held isothermally for 2 min at 50 °C, increased at a rate of 6 °C/min to 120 °C, then increased at a rate of 15 °C/min to 240 °C. The method of internal normalization was used for the quantitative analyses of the post-reaction mixtures.

## 3. Results and Discussion

### 3.1. Characterization of the Obtained Materials

In order to determine the textural properties (S_BET_, V_tot_, V_mic_) of the obtained materials, they were subjected to N_2_ sorption tests at −196 °C. Table 2 shows the parameters characterizing the porous structure and acid-site concentrations for activated carbons obtained from biomass.

The C_AC sample was characterized by the highest value of the specific surface area (1566 m^2^/g). This material also had the largest total pore volume of 0.694 cm^3^/g and a micropore volume of 0.540 cm^3^/g. For this sample, the volume of pores with a diameter of 0.73–1.0 nm was 0.123 cm^3^/g, and it was the highest among all tested samples. The volume of pores with a diameter of 1–2 nm was 0.139 cm^3^/g, and it was also the highest recorded value among the tested carbonaceous materials.

The S_AC sample showed the least developed surface among all obtained porous carbonaceous materials (S_BET_ = 1366 m^2^/g, V_tot_ = 0.584 cm^3^/g, V_mic_ = 0.477 cm^3^/g). The volume of pores with a diameter of 0.73–1.0 nm was 0.097 cm^3^/g, and the volume of pores with a diameter of 1–2 nm was 0.154 cm^3^/g.

The value of the specific surface area for the O_AC sample was 1416 m^2^/g, the total pore volume was 0.643 cm^3^/g, and the micropore volume was 0.482 cm^3^/g. The volume of pores with a diameter of 0.73–1.0 nm was 0.097 cm^3^/g, and the volume of pores with a diameter of 1–2 nm was 0.111 cm^3^/g. These results show the highly developed specific surface area and porosity of the biomass-based materials. Similar results for this type of precursor were obtained by Wang et al. [39] from coffee grounds (S_BET_ = 1525 m^2^/g, V_tot_ = 0.77 cm^3^/g, V_micro_ = 0.54 cm^3^/g), Fernandez et al. [28] from orange peels (S_BET_ = 1090 m^2^/g, V_tot_ = 1.2 cm^3^/g, V_micro_ = 0.2364 cm^3^/g), and Zou et al. [83] from sunflower seed hulls (S_BET_ =1908–2122 m^2^/g, V_tot_ = 1.023–1.627 cm^3^/g, V_micro_ = 0.2–0.742 cm^3^/g).

Acid-site concentration was determined using the acid–base titration method. The highest concentration of acid centers was recorded for the activated carbon from coffee grounds (0.50 mmol/g). Similar values were recorded for the activated carbon from sunflower husk (0.45 mmol/g). The carbonaceous material from orange peels was characterized by the lowest content of acid sites (0.25 mmol/g).

**Figure 1:**
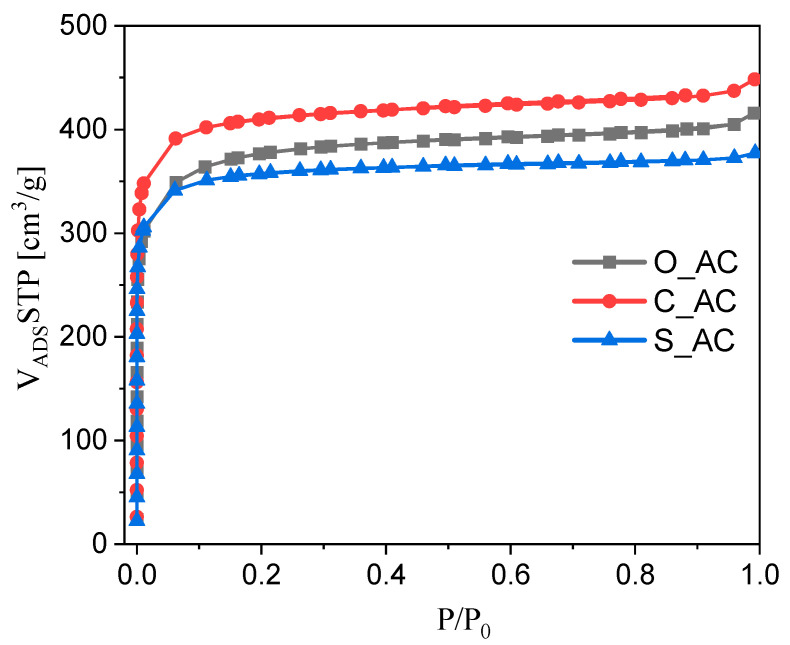
The sorption isotherms of N_2_ at −196 °C for tested activated carbons.

Figure 1 shows the sorption isotherms of N_2_ at −196 °C for tested activated carbons.

It can be seen from Figure 1 that all samples showed a rapid increase in the gas adsorption in the initial relative pressure range and the adsorption isotherm determined parallel to the P/P_0_ axis. This dependence proves that all activated carbons obtained from biomass are microporous materials [84]. All presented isotherms were reversible isotherms, for which no hysteresis loops were observed. The isotherms shown in Figure 1 correspond to class I isotherms as defined by the International Union of Pure and Applied Chemistry (IUPAC). Type I isotherms are characteristic for microporous materials [85]. Zhang et al. [86] and Yang et al. [87] also received similar I type isotherms which were characteristic for microporous materials.

**Figure 2:**
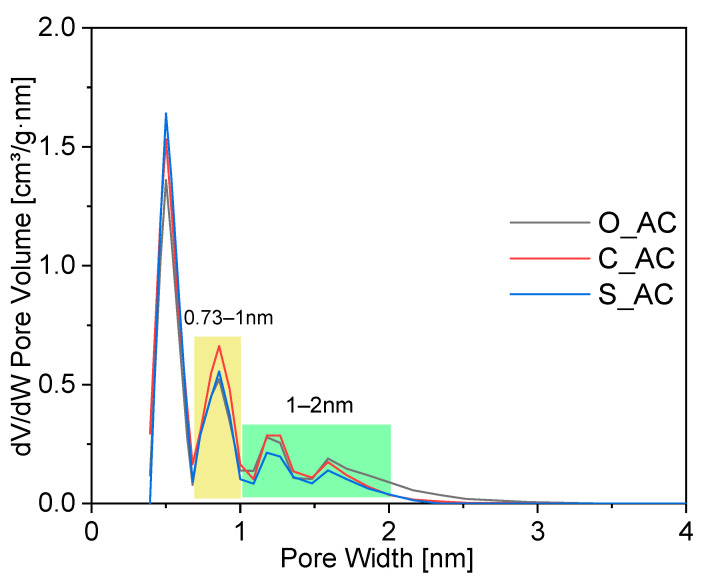
The pore volume distribution for tested activated carbons.

An analysis of the pore size distribution of activated carbons was also performed. Curves were established via the analysis of adsorption isotherms N_2_ at −196 °C. The pore distribution shown in Figure 2 confirms that the measured activated carbons, in addition to the micropores with a diameter of approximately 0.3 nm to 2 nm, had narrow mesopores (~2.1–2.7 nm) in their structure. Similar results were obtained by Serafin [88], Wei et al. [30], and Muniandy [89], where a high volume of micropores below 2 nm was also noted.

**Figure 3:**
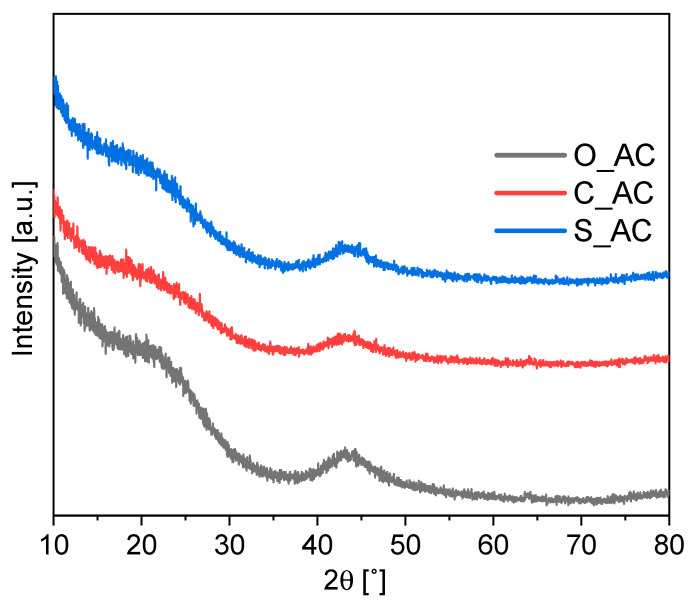
Diffractograms of activated carbons from biomass.

Figure 3 illustrates the X-ray diffraction profiles of the O_AC, C_AC and S_AC samples.

Diffractograms presented in Figure 3 show broad peaks, as well as the absence of a sharp peak. The absence of a sharp peak may indicate the amorphous structure of the materials. However, the occurrence of broad peaks (around 26° and 43°) showed signs of the formation of a crystalline carbonaceous structure, resulting in better layer alignment [90]. A similar result was proposed by Jiang et al. [91] for bituminous coal as a precursor, by Martínez-Casillas et al. [92] for activated carbon from pecan nutshell, and by Wei et al. [30] in studies concerning the synthesis of carbon materials from orange peels.

**Figure 4:**
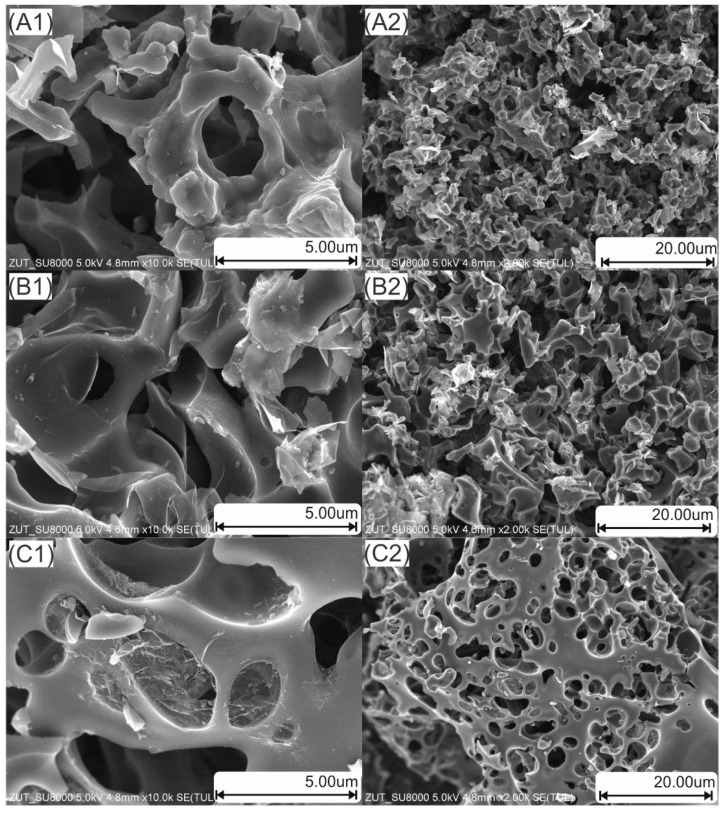
SEM micrographs of the activated carbons (**A1**,**A2**)—activated carbon from orange peels, (**B1**,**B2**)—activated carbon from coffee grounds, (**C1**, **C2**)—activated carbon from sunflower husks).

SEM micrographs of the tested activated carbons samples are shown in Figure 4.

The SEM images show an irregular surface morphology. The surface of all carbonaceous materials from biomass was characterized by a well-developed structure, as evidenced by the presence of holes of various diameters and shapes. The (A1), (A2), (B1) and (B2) micrographs show a very similar picture of carbonaceous materials. Irregularly shaped holes are visible. Micrographs (C1) and (C2) showing activated carbon from sunflower husks are characterized by holes of a regular, oval shape. The presence of a porous structure facilitates the diffusion process indispensable in the heterogeneous catalysis for transport of both reactants and products. Fernandez et al. [28] and Wei et al. [30] obtained the activated carbon from orange peel with a similar morphology. Carbonaceous materials from coffee grounds obtained by Goncalves [40] and Pagalan [93] also showed a morphology similar to the surface morphology of the activated carbon obtained from coffee grounds characterized in this work. Moreover, the morphology of activated carbon from sunflower was similar to that described in the article of Saleh et al. [50].

**Figure 5:**
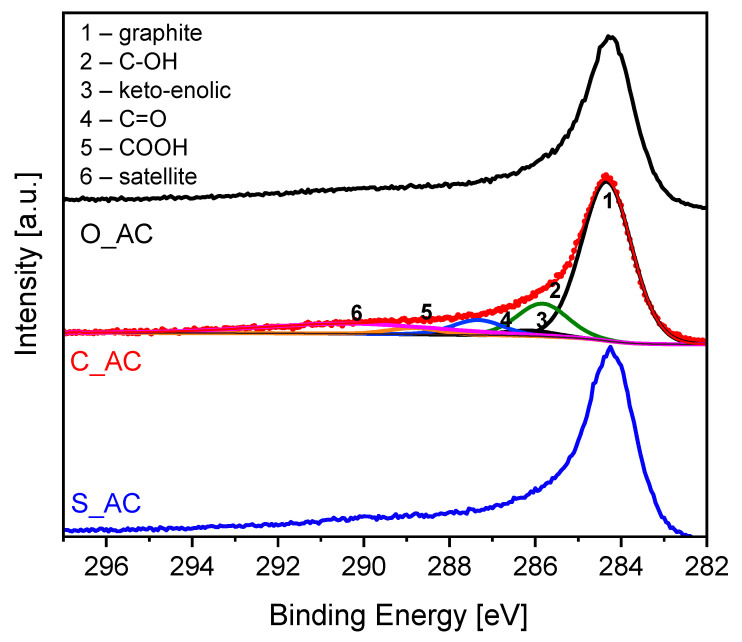
C 1*s* X-ray photoelectron spectra; the components are indicated for the C_AC sample.

The samples were characterized with the application of X-ray photoelectron spectroscopy (XPS). In Figure 5, the C 1*s* signals are presented. The deconvolution of the C 1*s* signal allowed the quantitative analysis of carbon functional groups (Table 3). The detailed procedure of C 1*s* deconvolution is described elsewhere [94].

The material obtained from sunflower husks contained the lowest content of C–O groups over the surface and the highest content of keto-enolic groups. The lowest content of keto-enolic groups characterized the material obtained from orange peels.

**Figure 6:**
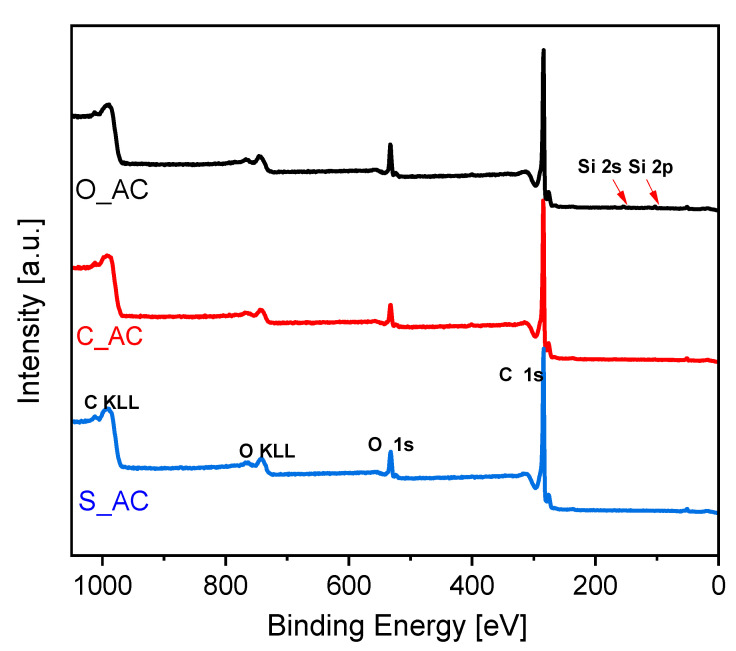
Survey X-ray photoelectron spectra.

Figure 6 presents the X-ray photoelectron spectra in the 0–1050 eV energy range. The main signals are indicated over the spectra.

The evaluation of the spectra enabled quantitative elemental analysis of the surface. The atomic concentrations of elements present over the surface are presented in Table 4.

In general, the elemental XPS analysis showed a high purity of obtained materials and low inorganic matter content over the surface. This is in line with XRD analysis where there was a lack of sharp reflexes typical for ash content (Figure 3). The XRF analysis showed the presence of other elements (Table 5). 

However, XRF delivers the average elemental bulk concentration, whereas XPS shows the elemental surface concentration from c.a. 1 nm depth. Moreover, all elements other than carbon and oxygen detected by XRF were below 1 wt.% content.

### 3.2. Activity of Activated Carbons

**Figure 7:**
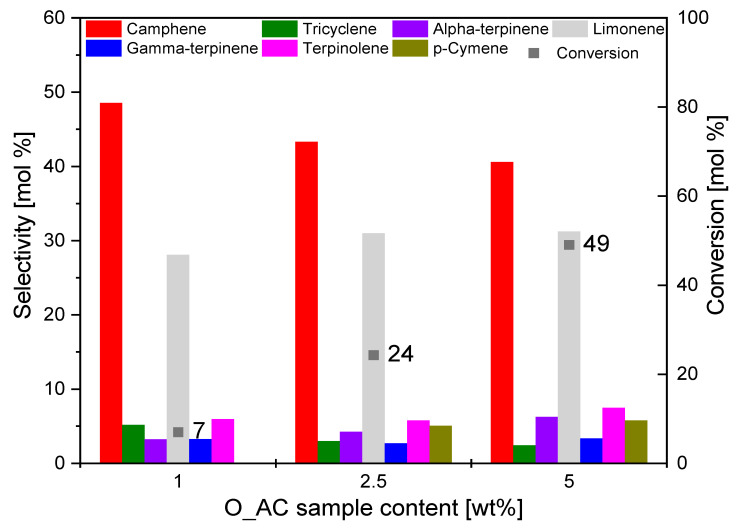
Influence of the O_AC sample content on the selectivity of the main products and conversion of organic raw material (alpha-pinene) after 3 h.

In the first stage of the studies on the catalytic activity of the obtained activated carbons, the influence of the content of the appropriate activated carbon in the reaction mixture on the values of conversion and selectivity of the main products of alpha-pinene isomerization was investigated. The studies on catalytic activity were carried out under the following conditions: reaction time 3 h, amount of alpha-pinene used in the reaction 3 g, and temperature 160 °C. Figure 7 shows the effect of the O_AC catalyst content on the conversion of alpha-pinene and selectivity of major products.

It can be seen from Figure 7 that the conversion of alpha-pinene increased with increasing content of O_AC catalyst in the reaction mixture. The highest value of the conversion of alpha-pinene was recorded for the catalyst content of 5 wt.%, and its was 49 mol.%.

The selectivity of the main reaction products (camphene and limonene) remained at a similar level across the whole range of studied catalyst content. We can say that the selectivity to camphene slightly decreased with increasing O_AC catalyst content in the reaction mixture (for O_AC sample content 1 wt.%, it amounted to 48 mol.%, for O_AC sample content 2.5 wt.%, it was 43 mol.%, and, for O_AC sample content 5 wt.%, it reached 41 mol.%).

As the content of the O_AC catalyst in the reaction mixture increased, the value of selectivity of limonene increased only slightly. The selectivity of limonene amounted to 28 mol.% for the catalyst content of 1 wt.%, 31 mol.% for catalyst content of 2.5 wt.%, and 32 mol.% for catalyst content of 5 wt.%. In addition to the main reaction products, the following products were formed in small amounts: tricyclene, terpinolene, gamma-terpinene, and alpha-terpinene. Furthermore, *p*-cymene was not formed at the catalyst content of 1 wt.%.

**Figure 8:**
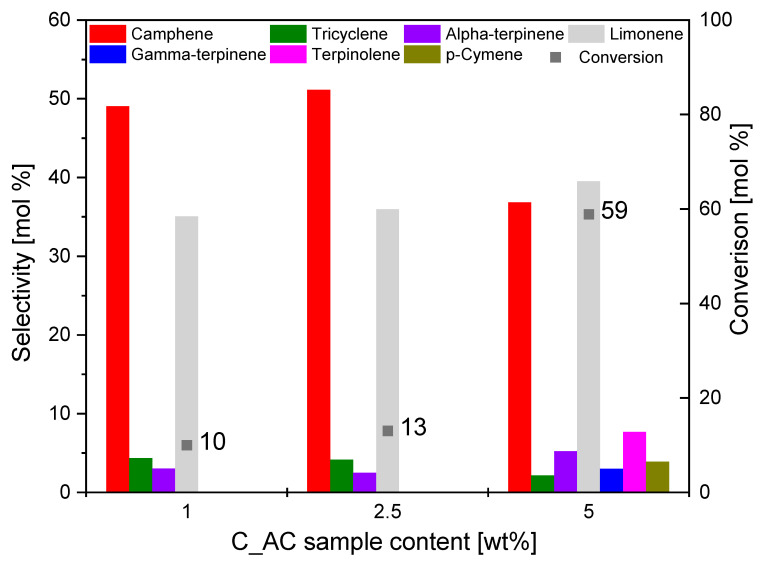
Influence of the C_AC sample content on the selectivity of the main products and conversion of organic raw material (alpha-pinene) after 3 h.

Figure 8 shows the effect of C_AC catalyst content on the conversion of the organic raw material (alpha-pinene) and selectivity of the main products.

It was noted that, with the increase in C_AC content in the reaction mixture, the conversion of alpha-pinene increased significantly (Figure 8). For the catalyst content of 5 wt.%, the conversion was 59 mol.%. This is because increasing the amount of catalyst increased the number of pores in which the isomerization reaction was most likely to occur, as described later. This allowed more alpha-pinene particles to enter in the pores and increased the conversion of this raw material. The highest value of selectivity of camphene was recorded for the catalyst content of 2.5 wt.%, while the value of the conversion of alpha-pinene for this content of catalyst was relatively low (13 mol.%). Selectivity to limonene for C_AC catalyst contents of 1 wt.% and 2.5 wt.% remained at a similar level and amounted to 30 mol.% and 31 mol.%, respectively, whereas, for the content of 5 wt.%, it was 40 mol.%. For all catalyst contents, the formation of compounds such as alpha-terpinene and tricyclene with lower selectivity was noted. At a content of 5 wt.%, *p*-cymene, terpinolene, and gamma-terpinene were also formed.

**Figure 9:**
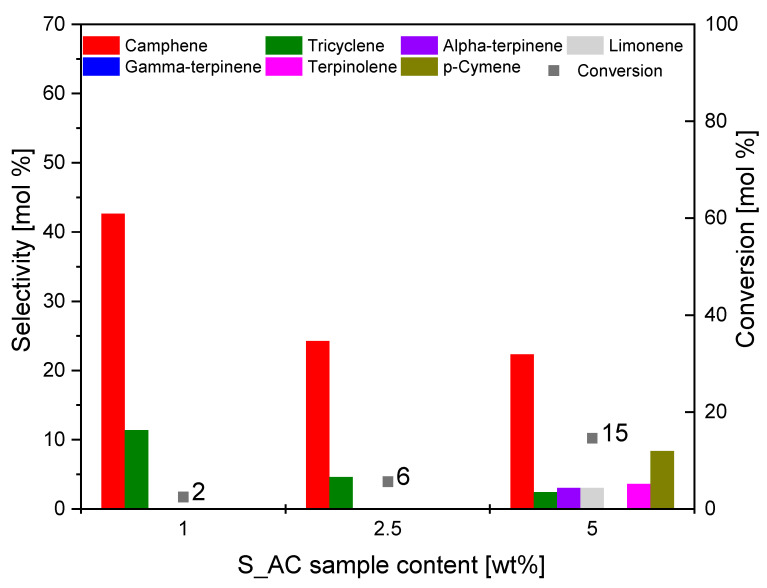
Influence of the S_AC sample content on the selectivity of the main products and conversion of organic raw material (alpha-pinene) after 3 h.

The effect of the catalyst content in the reaction mixture on the course of isomerization of alpha-pinene was also investigated for the S_AC sample. The results are shown in Figure 9.

It is visible from Figure 9 that increasing the S_AC catalyst content in the reaction mixture increased the value of conversion of alpha-pinene; for the content of the S_AC catalyst 5 wt.%, the alpha-pinene conversion was 15 mol.%. It was found that the conversion of alpha-pinene was at the limit of the measurement error, and that the activated carbon from sunflower husks was not active for contents of 1 wt.% and 2.5 wt.%. Only an increase in the catalyst content in the reaction mixture above 2.5 wt.% led to an increase in the conversion and selectivity to individual products.

For the S_AC catalyst content of 5 wt.%, the selectivity of camphene was 22 mol.%. The byproduct formed with the highest selectivity was *p*-cymene (8 mol.%). Limonene (3 mol.%), alpha-terpinene (3 mol.%), terpinolene (4 mol.%), and tricyclene (2 mol.%) were also formed, but with less selectivity.

The tested catalysts showed different activity in the process we studied. As shown in our earlier publication [72], during alpha-pinene isomerization, we can observe two main pathways: path A where polycyclic compounds are formed (mainly camphene and tricyclene) and path B where monocyclic compounds (mainly limonene and terpinolene) are formed, whereby limonene and terpinolene can subsequently be isomerized or dehydrogenated. For the O_AC catalyst, we could observe both paths of transformation, with path A being the dominant one. Monocyclic products were formed in smaller amounts, but they were formed at each of the tested catalyst contents. On the other hand, *p*-cymene (the product of dehydration of these compounds) was formed only at the two highest catalyst contents. The results obtained with the C_AC catalyst were very similar in terms of the direction of transformation of alpha-pinene. However, only the application of the highest catalyst content allowed obtaining isomerization products of monocyclic compounds, as well as p-cymene. In the case of the S_AC catalyst, the test results indicate that path A of the alpha-pinene transformation was preferred, wherein camphene and tricyclene were formed with a slightly lower overall selectivity (about 5 mol.%) than in studies with previous catalysts. On the other hand, no limonene formation was observed; only with a catalyst content of 5 wt.% were only small amounts of limonene and terpinolene isomerization products detected in the post-reaction mixture. As with the previous two samples, *p*-cymene formation was only observed for the highest catalyst content in the reaction mixture. In research on this catalyst, the significantly lower alpha-pinene conversion compared to other catalysts (more than threefold) was also noticeable. Summarizing the research on the influence of the amount of the catalyst on the course of alpha-pinene isomerization, it can be said that, for all tested activated carbons, the content of 5 wt.% was the most advantageous.

In the next stage of the studies, the influence of temperature on the values of conversion of alpha-pinene and selectivity of products of the alpha-pinene isomerization process was investigated. For this purpose, the catalytic tests were carried out under the following conditions: reaction time 3 h, amount of alpha-pinene used in the reaction 3 g, content of catalyst 5 wt.%, and temperature 140, 160, and 180 °C.

**Figure 10:**
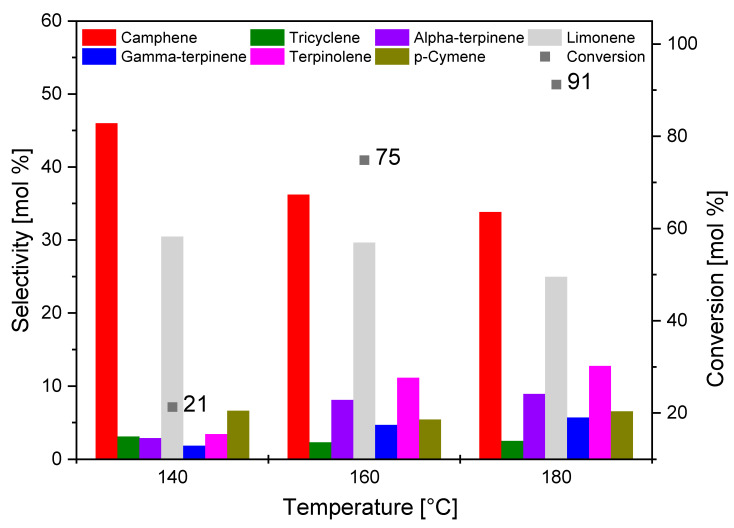
Comparison of the selectivity of the main products and the conversion of organic raw material (alpha-pinene) at different temperatures after 3 h for the O_AC sample.

Figure 10 shows the effect of temperature on the isomerization process carried out in the presence of O_AC catalyst.

It is visible from Figure 10 that the increase in temperature caused a marked increase in the value of conversion of alpha-pinene (21 mol.% for 140 °C, 75 mol.% for 160 °C, and 91 mol.% for 180 °C). This is due to the fact that increasing the temperature increased the diffusion rate of alpha-pinene molecules into the pores where the isomerization process took place, which in turn increased the conversion of this organic raw material.

It was noted that, as the reaction temperature increased, the values of selectivity of the main products decreased (from 41 mol.% to 34 mol.% for camphene and from 30 mol.% to 25 mol.% for limonene). The decrease in selectivity of the main reaction products may be due to the fact that, with increasing temperature, an increase in the selectivity of transformation to byproducts of isomerization was visible. These byproducts were terpinolene, gamma-terpinene, *p*-cymene, and alpha-terpinene.

**Figure 11:**
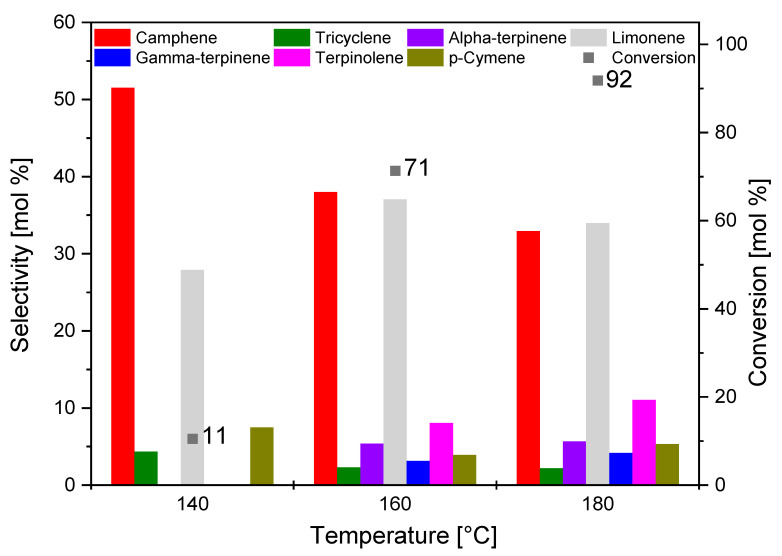
Comparison of the selectivity of the main products and the conversion of organic raw material (alpha-pinene) at different temperatures after 3 h for the C_AC sample.

A comparison of the selectivity of the main products and the conversion of alpha-pinene using the C_AC catalyst at different temperatures is shown in Figure 11.

Figure 11 shows that, with the increase in temperature, the conversion of alpha-pinene in the presence of C_AC catalyst increased to the maximum value of 92 mol.% at the temperature of 180 °C. The main reaction products, which were formed with similar selectivity at 160 and 180 °C, were camphene (35–38 mol.%) and limonene (35–37 mol.%). At 140 °C, the slightly higher selectivity of camphene and low conversion of alpha-pinene (11 mol.%) indicated a low reaction rate.

**Figure 12:**
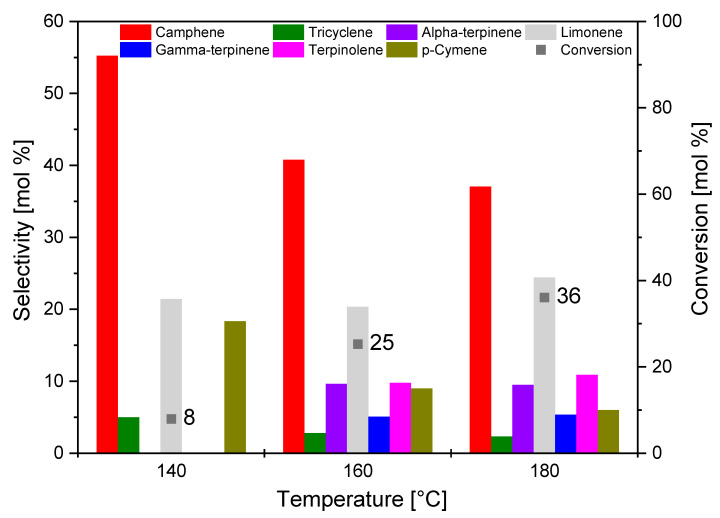
Comparison of the selectivity of the main products and the conversion of organic raw material (alpha-pinene) at different temperatures after 3 h for the S_AC sample.

A comparison of the selectivity of the main products and the conversion of organic raw material (alpha-pinene) using the S_AC catalyst at different temperatures is shown in Figure 12.

The conversion of alpha-pinene for the reaction carried out at the temperature of 140 °C amounted to 8 mol.% (Figure 12). It can be concluded that the process temperature of 140 °C was too low, and the substrate reacted in a small amount. When using activated carbon from sunflower husks as the catalyst, the selectivity of camphene and *p*-cymene decreased with increasing temperature, while the selectivity of limonene and other products (terpinolene, gamma-terpinene, and alpha-terpinene) increased slightly.

Comparing the studies of the influence of temperature on the results obtained with the three tested catalysts, it can be said that the C_AC and S_AC catalysts were characterized by path A of alpha-pinene transformation being preferred at the lowest temperature. At this lowest temperature, the formation of *p*-cymene was also observed, which proves that the products formed along the path A were immediately transformed into *p*-cymene. Products produced according to path B appeared only at the two highest tested temperatures. Summarizing the studies on the influence of temperature on the course of alpha-pinene isomerization, the temperature of 160 °C was considered the most favorable for all tested catalyst samples. This is due to the fact that, at 180 °C, alpha-pinene boiled strongly and the selectivity to camphene and limonene decreased. This was caused by the subsequent reactions where limonene isomerized, and other products dimerized and polymerized. Further studies on the influence of reaction time on the course of isomerization and its kinetics were carried out at the temperature of 160 °C.

**Figure 13:**
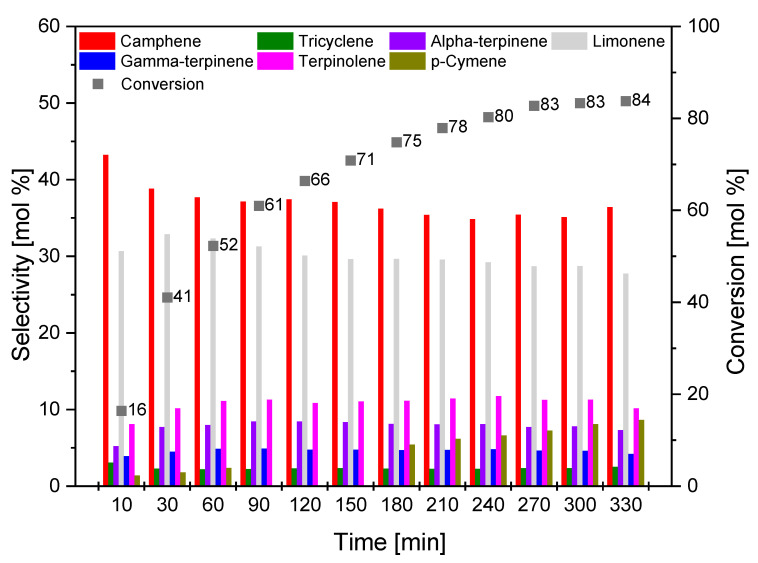
Influence of time on the selectivity of the main products and conversion of organic raw material (alpha-pinene) for O_AC sample.

**Figure 14:**
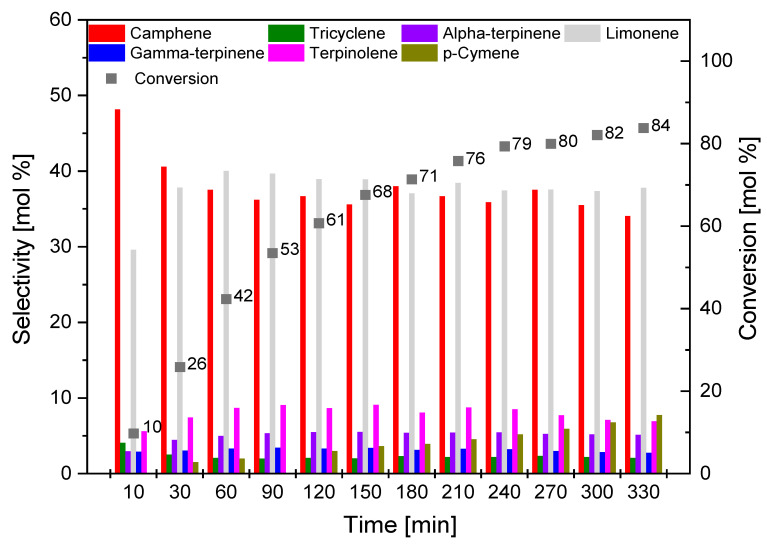
Influence of time on the selectivity of the main products and the conversion of organic raw material (alpha-pinene) for C_AC sample.

**Figure 15:**
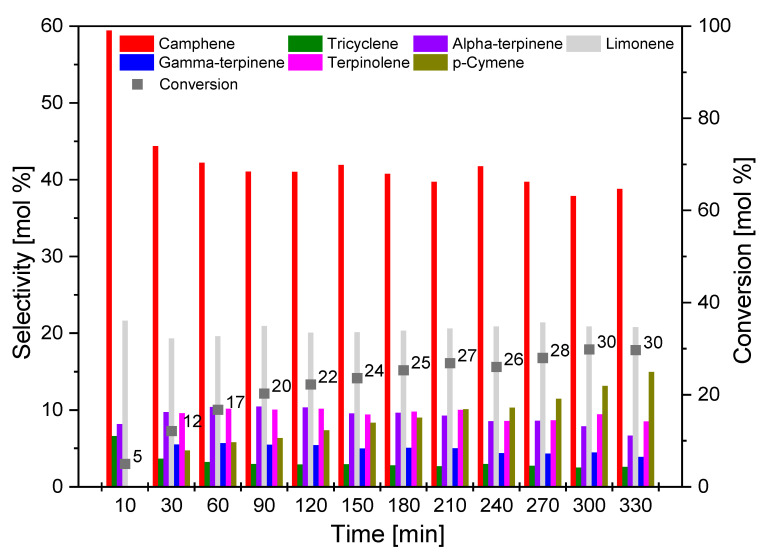
Influence of time on the selectivity of the main products and the conversion of organic raw material (alpha-pinene) for S_AC sample.

Figure 13, Figure 14 and Figure 15 show the influence of reaction time on the course of isomerization of alpha-pinene at 160 °C. For the reaction time effect study, 8 g of alpha-pinene and 0.4 g (5 wt.%) of the appropriate catalyst (O_AC, C_AC, S_AC) was used. The test mixtures were collected in the range of 10–330 min at 30 min intervals.

For catalyst O_ AC (Figure 13), the selectivity of camphene was the highest for reaction time of 10 min, while the conversion was relatively low (16 mol.%). Over the duration of the process of isomerization, the selectivity to camphene decreased from 47 mol.% (time 10 min) to 36 mol.% (time 300 min). The selectivity of limonene was the highest for the reaction time of 30 min and amounted to 33 mol.%, decreasing to 27 mol.% (reaction time 330 min). The highest value of the conversion of alpha-pinene was obtained for a reaction time of 330 min, and its was 84 mol.%.

The isomerization carried out in the presence of the catalyst obtained from coffee grounds (C_AC; Figure 14) followed a similar course to the catalyst obtained from orange peels (O_AC). The selectivity of camphene was the highest for a reaction time of 10 min, while the conversion of alpha-pinene was low (10 mol.%). The selectivity of camphene decreased with prolongation of the reaction time; for the reaction time of 330 min, it amounted to 34 mol.%. The selectivity of limonene remained similar throughout the reaction time studied. The highest value of the conversion of alpha-pinene for C_AC sample was obtained for a reaction time of 330 min, and it was 84 mol.%.

Among all the activated carbons obtained from biomass, the activated carbon obtained from sunflower husks (S_AC; Figure 15) was characterized by the weakest activity in the studies on the influence of reaction time. The maximum conversion of alpha-pinene was achieved for a reaction time of 300 min (30 mol.%). The selectivity of limonene remained similar throughout the studied reaction time and did not exceed 23 mol.%. The selectivity of camphene at the highest conversion of alpha-pinene was 38 mol.%.

The study of the influence of temperature showed that, despite the prolongation of the reaction time for the O_CA sample, the preferred direction of transformation was path A. In the case of the C_AC catalyst, the change of the privileged reaction to path B for reaction times over 30 min was noticeable (a higher total selectivity of the transformation to limonene and terpinolene with respect to camphene and tricyclene). In the case of sample 3 (S_AC), it is characteristic that path A was preferred to path B, and that the amount of *p*-cymene produced in the reaction was significantly increased (this was especially noticeable when comparing the results for the S_AC sample with the results for the O_AC sample). Summarizing the studies on the influence of reaction time on the course of alpha-pinene isomerization for all tested catalyst samples, the time of 3 h was considered the most favorable. This time allowed obtaining high selectivity of camphene and limonene with relatively high alpha-pinene conversion.

**Figure 16:**
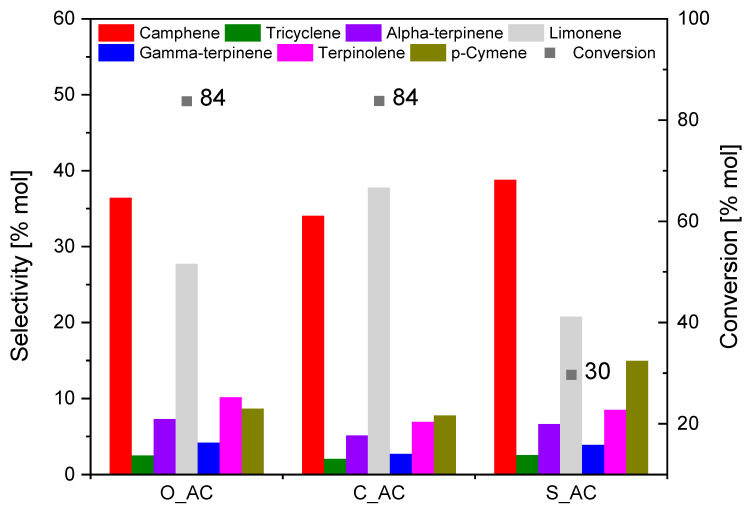
Comparison of the catalytic activity of activated carbons at the most favorable isomerization conditions (content of catalyst 5 wt.%, temperature 160 °C, and reaction time 3 h).

Figure 16 shows a comparison of the catalytic activity of carbonaceous materials obtained from biomass. The most favorable conditions for all catalysts were as follows: content of catalyst 5 wt.%, temperature 160 °C, and reaction time 3 h.

According to Figure 16, the most active catalyst in the isomerization process was activated carbon obtained from coffee grounds (C_AC), because the sum of selectivity of the main products (camphene and limonene) for the C_AC sample was the highest (72 mol.%). For this catalyst, the conversion of alpha-pinene was 84 mol.%. Slightly worse results were obtained for the O_AC catalyst, with a comparable conversion of alpha-pinene but a lower sum of selectivity of camphene and limonene (64 mol.%).

On the other hand, activated carbon obtained from sunflower husks (S_AC) was characterized by the lowest catalytic activity, which makes it an unfavorable catalyst for the alpha-pinene isomerization process. The conversion of alpha-pinene for the S_AC material was only 30 mol.%, and the sum of selectivity of camphene and limonene amounted to 60 mol.%.

Comparing the textural properties of the three tested catalysts (presented in Table 2), it can be seen that the C_AC sample was characterized by the largest specific surface area (1566 m^2^/g) and the largest micropore volume (0.540 cm^3^/g) among the tested catalysts. Smaller values of these two values were obtained for the sample designated as O_AC (1416 m^2^/g and 0.482 cm^3^/g). Additionally, the proportion of mesopores in the O_AC sample was largest for the apparent pore range of 2.7 nm. The values obtained for the S_AC sample significantly differed from the values obtained for the other two samples. This difference in the textural parameters characterizing the tested three samples of activated carbons probably led to the carbons obtained from coffee grounds and orange peels being the most active.

Taking into account the pore size of the tested samples (Figure 2, Table 2), i.e., 0.4–0.7 nm, 0.7–1.0 nm, 1.1–1.4 nm, and 1.5–2.5 nm, and the number of pores, we can conclude that the C_AC and O_AC samples had the largest number of pores with a size of 0.7–1.0, 1.1–1.4 nm and 1.5–2.5 nm, whereas the S_AC sample had the largest number of pores with a size of 0.4–0.7 nm. The size of the alpha-pinene molecule is about 0.7 nm [95]; thus, pores with a size of 0.7–1.0 and 1.1–1.4 nm or larger are of greatest importance for the isomerization process of this compound. The results in Figure 2, thus, confirm that the catalyst samples C_AC and O_AC allowed diffusion of alpha-pinene molecules into the interior of the pores, where the investigated reaction took place, and that these catalyst samples should be the most active in the process. In addition, the results presented in Table 2 for pore volumes of 0.73–1 nm and 1–2 nm for the O_AC and C_AC samples show that C_AC activated carbon had a greater number of pores with a pore size of 0.73–1 nm (volumes of 0.123 cm^3^/g and 0.097 cm^3^/g, respectively), which probably determined the slight difference in the activity of O_AC and C_AC catalysts, in favor of the latter.

The acidity studies summarized in Table 2 indicate that, in the case of alpha-pinene isomerization, surface acidity did not affect the final activity of the catalyst, as the highly acidic sample of S_AC turned out to be inactive. The answer to why the catalyst sample obtained from sunflower husk was the least active may be reflected in the results of XRF tests. Therefore, the instrumental tests of the catalysts were supplemented with XRF tests. The results of these studies are presented in Table 5.

Table 5 shows that the tested samples of carbon materials differed significantly in the content of S, Cl, and K. The C_AC sample was characterized by a significantly higher content of Cl and K compared to the O_AC sample. It can be initially assumed that the abovementioned elements, most likely K^+^ and Cl^−^ ions, present in the pores of the tested active carbons, are of key importance for the alpha-pinene isomerization process. However, this requires further research.

### 3.3. Determination of the Kinetics Parameters

The overall kinetic studies of α-pinene isomerization over three activated carbons was performed for several order reactions, using a parameter estimation software based on the following equations:

First-order reaction:

(1) $$-\frac{{\mathrm{dC}}_{\alpha -\mathrm{pinene}}}{\mathrm{dt}}{=\mathrm{kC}}_{\alpha -\mathrm{pinene}},$$

Different reaction orders:

(2) $$\frac{C_{A}^{1-n}{-C}_{A_{0}}^{1-n}}{n-1}=\mathrm{kt},$$

where C*_α_*_-pinene_ is the alpha-pinene concentration, t is the reaction time, and k is the reaction rate constant.

According to the regression coefficients, it was concluded that α-pinene isomerization is a first-order reaction. This reaction order followed our early studies obtained for α-pinene isomerization over Ti_3_C_2_ and ex- [96] and clinoptilolite (modified with 0.1 M H_2_SO_4_—CLIN 0.1) [66]. Similar results were also evidenced by other authors, e.g., Ünveren et al. [65] and Allahverdiev et al. [97].

The determined reaction rate coefficients for the first-order reaction are compiled in Table 6.

The reaction rate constant calculated for α-pinene isomerization over the activated carbons derived from coffee grounds achieved the highest value equal to 0.3311 h^−1^, which is over sixfold higher than the calculated constant calculated for α-pinene isomerization over the activated carbons obtained from sunflower husks (k = 0.47 h^–1^).

The reaction network of the suggested mechanism of α-pinene isomerization over the activated carbons was the same as that over clinoptilolite. The advanced network of α-pinene isomerization is shown in Table 7.

An accurate reaction mechanism is a crucial element of reliable modeling. The advanced reaction mechanism was expressed by eight reaction routes, compiled in columns. Chemical equations of the basic and intermediate steps, including reactants and surface species, were placed in 17 rows.

Even when information is available, identifying the relevant data from the variety of numbers can be a difficult process without help. Thus, in order to explain the significance of 1, 0, and −1 values, a brief explanation is provided for Table 7: 1 is connected with the appearance of the order of a basic reaction which must lead from reactants to products, e.g., **1** (denoted by bold and underlined font at the crossing of 3 row and 5 column) indicates that α-pinene leads to terpinolene just when it is maintained by an irreversible arrangement of Z·(α-pinene)_2_ from Z·(α-pinene); 0 indicates that a reaction equation described in a row is not interconnected with a product placed in a column, e.g., **0** (bold and underlined font at the crossing of 10 row and 1 column) implies that this is unlikely to lead to tricyclene from Z·(α-pinene)_2_ or (α + γ-terpinene) because both pathways are not connected; −1 relates to a reaction wherein an intermediary product is utilized as a final product in one step, e.g., **−1** (bold and underlined font at the intersection of 13 row and 8 column) signifies that *p*-cymene as a final product is produced in one step from terpinolene (intermediate product).

Completed kinetic modeling with the detailed derivation and parameter estimation for a system of differential equations was developed. However, in this paper, we present just some of the results. We rearranged the kinetic equations (a system of differential reactions directly incorporating time-dependent concentrations) for an analysis of selectivity dependence. Parameter calculation was achieved similarly to [65] utilizing Miniwork software. The system of differential Equations (3)–(6) was combined at each minimization step with the following aim function: sum of squares of determined and empirical values of mole fraction.

(3) $$-\frac{dC_{B}}{dC_{A}}=f_{1}+f_{2}\frac{C_{C}}{C_{A}}-f_{3}\frac{C_{B}}{C_{A}}.$$

(4) $$-\frac{dC_{C}}{dC_{A}}=f_{4}-f_{2}\frac{C_{C}}{C_{A}}+f_{3}\frac{C_{B}}{C_{A}}.$$

(5) $$-\frac{dC_{D}}{dC_{A}}=f_{5}-f_{6}\frac{C_{D}}{C_{A}}.$$

(6) $$-\frac{dC_{G}}{dC_{A}}=f_{7}\frac{C_{D}}{C_{A}}+f_{8}\frac{C_{E}}{C_{A}}+f_{9}\frac{C_{F}}{C_{A}}.$$

For subsequent modeling, one assumption was proposed: the rates corresponding to camphene and tricyclene (1→2→5→7; 1→2→4→6), as well *p*-cymene (3→8→11→14; 3→9→12→14; 3→10→13→14), are not related to each other.

Studies of constants from a physical chemistry perspective for such a complex model only have meaning when kinetic parameters can be modeled separately. Accordingly, to get worthy values of parameters in a specific case of α-pinene isomerization, it is useful to manage the isomerization reactions of similar substances. Generally, values of kinetic constants, calculated from such independents experiments, should be combined in the general model, described by Equations (3)–(6). Nevertheless, for reaction modeling, good specification of experimental data allows one to use this model even with the dimensionless parameters collected in Table 8.

The values of the dimensionless parameters in Equations (3)–(6) were obtained for α-pinene isomerization over the most efficient activated carbon derived from coffee grounds, and the results are compiled in Table 8 with their standard errors.

The suggested model and mechanism were suitable with the experimental data. The values of the experimental and calculated data were compared; overall, a good fit was achieved. Calculated empirical dimensionless parameters for α-pinene isomerization over the activated carbons from coffee grounds achieved in current studies were different from values in our previous studies over clinoptilolite [66]. However, the dependency amongst all parameters was maintained. Moreover, it was confirmed that the α-pinene isomerization is a first-order reaction over all activated carbons. However, it was evidenced that the α-pinene isomerization rate was different, achieving the highest value over activated carbons derived from coffee grounds.

## 4. Conclusions

In summary, activated carbons from different biomass precursors (orange peels, coffee grounds, and sunflower husks) were prepared successfully by chemical activation using potassium hydroxide. The obtained carbonaceous materials were characterized by a well-developed specific surface area ranging from 1366 m^2^/g to 1566 m^2^/g, a total pore volume ranging from 0.584 cm^3^/g to 0.694 cm^3^/g, and microporous structure ranging from 0.477 cm^3^/g to 0.54 cm^3^/g. XRD analysis proved the amorphous structure of the prepared materials. SEM spectroscopy of analyzed samples showed an irregular surface morphology. XPS and XRF spectroscopy showed high purity with trace amounts of inorganic matter content over the surface of obtained carbon materials.

Activated carbons obtained from waste biomass turned out to be active catalysts for the alpha-pinene isomerization process. Among the three tested samples, two were distinguished by particularly high activity in the isomerization process: activated carbons obtained from coffee grounds and orange peels. Such high activity of these two materials most probably resulted from their large specific surface area, as well as from their high content of pores with a diameter of 0.7–1.0, 1.1–1.4, and 1.5–2.5 nm. Pores with the first diameter range seem to be particularly significant for the course of the isomerization process; therefore, the activated carbon obtained from the coffee grounds, which had a larger number of pores in this diameter range compared to the activated carbon obtained from orange peels, was more active in alpha-pinene isomerization. The presence of potassium and chlorine ions in the pores may also be of key importance for the alpha-pinene isomerization process, but this requires further research. Differences in the direction of transformations were observed for the tested catalysts. Time effect studies showed that path A (the pathway to camphene and tricyclene formation) was favored for the catalysts obtained from orange peels and sunflower husk. For the catalyst obtained from coffee grounds, path B, i.e., the formation of monocyclic products (limonene, terpinolene), prevailed. In this case, the size of the product molecule was potentially of great importance for the proper orientation of alpha-pinene transformation. In the case of the catalyst obtained from sunflower husk, almost threefold lower alpha-pinene conversion and significantly higher selectivity of the transformation to *p*-cymene were noticeable.

Generally, for all tested catalysts, the same favorable conditions for carrying out the alpha-pinene isomerization process were determined (temperature 160 °C, amount of catalyst 5 wt.%, and reaction time 3 h). However, in the case of the sample of activated carbon obtained from sunflower husk, research should be carried out to increase the activity of this sample, e.g., by impregnating its surface with metals. Taking into account the alpha-pinene conversion, the results obtained on the C_AC catalyst (the most active among those tested) were very similar to the results obtained on the W_2_O_3_–Al_2_O_3_ catalyst and slightly lower than those on the modified Al or Ti mesoporous silicates and on the carbon obtained from coffee grounds described in the literature (Table 1). On the other hand, the selectivity of transformation to limonene obtained on this catalyst was very high and close to the best results described in the literature for catalysts such as HCl-modified clinoptilolite and activated carbon from coffee grounds. The selectivity of the transformation to camphene was similar to the results obtained on zeolites, modified silicates, and activated carbon from coffee grounds.

The proposed method of obtaining activated carbons from waste biomass and the way of using these carbons as catalysts for the alpha-pinene isomerization process (the compound obtained from raw materials of natural origin) are examples of an effective way of processing natural resources into materials and compounds with large applications. It should also be emphasized at this point that such raw materials are renewable and have a high availability and relatively low price. This way of obtaining the activated carbons is very simple and requires the use of only two reagents. Thus, this method is cheap, and it does not generate much chemical waste.

Products obtained by alpha-pinene isomerization (camphene and limonene) have numerous applications, both in medicine and in the cosmetics and food industries, as well as in organic synthesis and in the production of polymers. Hence, taking into account the use of cheap activated carbons obtained from waste biomass and obtaining such valuable products of alpha-pinene transformation, this method of alpha-pinene isomerization should be further researched and developed.

## Figures and Tables

**Table 1:** Isomerization of alpha-pinene: conversion and selectivity using selected catalysts.

Catalyst	Conversion of Alpha-Pinene (mol.%)	Selectivity of Camphene (mol.%)	Selectivity of Limonene (mol.%)	Ref.
HCl-modified clinoptilolite	41	57	32	[65]
SO_4_/Al_x_ZrO_2_	32	¯	¯	[82]
W_2_O_3_–Al_2_O_3_	73	55	¯	[78]
Fe-loaded clinoptilolite	100	38	2	[69]
Cr-loaded clinoptilolite	100	24	11	[69]
H_2_SO_4_-modified clinoptilolite from Turkey	18	46	19	[66]
Calcined natural zeolites	100	32	25	[75]
Al-MCM-41	98	30	30	[70]
Ti-MCM-41	98	35	21	[71]
Ti-SBA-15	98	24	24	[72]
Activated carbon from coffee grounds	84	34	38	In this study

**Table 2:** Surface properties of tested activated carbons.

Sample	S_BET_ (m^2^/g)	V_tot_ (cm^3^/g)	V_mic_ (cm^3^/g)	V_0.73–1 nm_ (cm^3^/g)	V_1–2 nm_ (cm^3^/g)	Acid-Site Concentration (mmol/g)
O_AC	1416	0.643	0.482	0.097	0.154	0.25
C_AC	1566	0.694	0.540	0.123	0.139	0.50
S_AC	1366	0.584	0.477	0.097	0.111	0.45

**Table 3:** The content of C 1*s* components expressed as atomic concentrations.

Assignment	O_AC	C_AC	S_AC
C	62.6	62.7	63.6
C–O	13.0	12.9	11.7
Keto-enolic	1.6	2.2	2.8
C=O	5.9	5.9	6.3
COOH	2.4	2.5	2.7
Satellite	14.6	13.9	12.8

**Table 4:** The atomic concentrations of elements present over the surface of the obtained carbon materials.

Sample	O 1*s*	C 1*s*	Si 2*p*
O_AC	7.43	91.49	1.08
C_AC	5.89	93.78	0.33
S_AC	6.6	93.4	0

**Table 5:** Results obtained by XRF method for activated carbons obtained from orange peels, coffee grounds, and sunflower husks.

Sample	Wt.%				
	Si	S	Cl	K	Ca
O_AC	0.264	0.416	0.182	0.595	0.289
C_AC	0.268	0.244	0.696	0.997	0.000
S_AC	0.246	0.418	0.155	0.225	0.280

**Table 6:** The α-pinene isomerization kinetic parameters.

Sample	k (h^−1^)	*R^2^*
O_AC	0.3178	0.9309
C_AC	0.3311	0.9670
S_AC	0.0573	0.9049

**Table 7:** Reaction mechanism for α-pinene isomerization.

No.	Steps	α-Pinene = Tricyclene	α-Pinene = Camphene	α-Pinene = Limonene	α-Pinene = α+γ-Terpinene	α-Pinene = Terpinolene	Limonene = *p*-Cymene	α+γ-Terpinene = *p*-Cymene	Terpinolene = *p*-Cymene
1	Z + A Ξ Z·(A)	1	1	1	1	1	0	0	0
2	Z·(A) ⇒ Z·(A)_1_	1	1	0	0	0	0	0	0
3	Z·(A) ⇒ Z·(A)_2_	0	0	1	1	1	0	0	0
4	Z·(A)_1_ ⇔ Z·(B)	1	0	0	0	0	0	0	0
5	Z·(A)_1_ ⇔ Z·(C)	0	1	0	0	0	0	0	0
6	Z·(B) Ξ Z + B	1	0	0	0	0	0	0	0
7	Z·(C) Ξ Z + C	0	1	0	0	0	0	0	0
8	Z·(A)_2_ ⇒ Z·(D)	0	0	1	0	0	0	0	0
9	Z·(D) Ξ Z + D	0	0	1	0	0	−1	0	0
10	Z·(A)_2_ ⇒ Z·(E)	0	0	0	1	0	0	0	0
11	Z·(E) Ξ Z + (E)	0	0	0	1	0	0	−1	0
12	Z·(A)_2_ ⇒ Z·(F)	0	0	0	0	1	0	0	0
13	Z·(F) Ξ Z + F	0	0	0	0	1	0	0	−1
14	Z·(D) ⇒ Z·(G)	0	0	0	0	1	0	0	0
15	Z·(E) ⇒ Z·(G)	0	0	0	0	0	0	1	0
16	Z·(F) ⇒ Z·(G)	0	0	0	0	0	0	0	1
17	Z·(G) Ξ G	0	0	0	0	0	0	0	1

Note. A—α-pinene, B—tricyclene, C—camphene, D—limonene, E—α + γ−terpinene, F—terpinolene, G—*p*-cymene, Z surface sites.

**Table 8:** Statistical parameters.

Dimensionless Parameter	Estimated Value	Standard Error (±)
f_1_	0.04	0.0036
f_2_	0.05	0.0011
f_3_	0.08	0.0041
f_4_	0.38	0.0278
f_5_	0.57	0.0294
f_6_	0.19	0.0165
f_7_	0.05	0.0077
f_8_	0.05	0.0082
f_9_	0.04	0.0063

## Data Availability

Not applicable.
